# Interpersonal synchrony across vocal and lexical modalities in interactions involving children with autism spectrum disorder

**DOI:** 10.1121/10.0013421

**Published:** 2022-09-08

**Authors:** Rimita Lahiri, Md Nasir, Manoj Kumar, So Hyun Kim, Somer Bishop, Catherine Lord, Shrikanth Narayanan

**Affiliations:** 1 Signal Analysis and Interpretation Laboratory, University of Southern California, Los Angeles, California 90089, USA; 2 Microsoft Artificial Intelligence for Good Research Lab, Redmond, Washington 98052, USA; 3 Amazon Alexa Artificial Intelligence, Cambridge, Massachusetts 02142, USA; 4 Center for Autism and the Developing Brain, Weill Cornell Medicine, New York, New York 10065, USA; 5 Department of Psychiatry, University of California, San Francisco, California 94143, USA; 6 Semel Institute of Neuroscience and Human Behavior, University of California, Los Angeles, California 90024, USA rlahiri@usc.edu, mdnasir@microsoft.com, manojpamk@gmail.com, sok2015@med.cornell.edu, bishop.somer@ucsf.edu, CLord@mednet.ucla.edu, shri@ee.usc.edu

## Abstract

Quantifying behavioral synchrony can inform clinical diagnosis, long-term monitoring, and individualised interventions in neuro-developmental disorders characterized by deficit in communication and social interaction, such as autism spectrum disorder. In this work, three different objective measures of interpersonal synchrony are evaluated across vocal and linguistic communication modalities. For vocal prosodic and spectral features, dynamic time warping distance and squared cosine distance of (feature-wise) complexity are used, and for lexical features, word mover's distance is applied to capture behavioral synchrony. It is shown that these interpersonal vocal and linguistic synchrony measures capture complementary information that helps in characterizing overall behavioral patterns.

## 1. Introduction

Autism spectrum disorder (ASD) (Baio, 2018; Maenner *et al.*, 2021) is a developmental condition primarily characterised by differences in social communication skills and abilities along with restricted repetitive behavior, interests, and movements. Prevalence of ASD in children in the United States has steadily risen from 1 in 150 in 2002 to 1 in 44 in 2021. ASD is a spectrum disorder with wide individual heterogeneity. Human-centered technological advances offer promise for supporting new evidence-driven possibilities in support of behavioral stratification as well as diagnosis and personalized treatment (Bone *et al.*, 2017; Narayanan and Georgiou, 2013).

In the recent years, computational approaches using signal processing and machine learning have been proposed for both research and clinical translation in mental health (Bone *et al.*, 2017). Computational methods have shown promise in supporting the diagnostic efforts by identifying the essential nosological modules, eliminating redundancy without compromising on accuracy (Bone *et al.*, 2016; Bone *et al.* 2015b; Kumar *et al.*, 2016). Computational techniques have also provided tools to further scientific understanding of interaction mechanisms. For instance, Bone *et al.* (2015a) connects objective signal-derived descriptors of vocal prosody to subjective perceptions of prosodic *awkwardness* and reports differences in acoustic–prosodic features between ASD and neurotypical individuals, including demonstrating interaction pattern differences in vocal prosody coordination that varied in accordance with a child's ASD symptom severity (Bone *et al.*, 2014a).

Synchrony (Johnson and Jacob, 2000; Nasir *et al.*, 2019) in interactions can be broadly viewed as an individual's reciprocity, coordination, or adaptation to other participants(s) in, and during, the interaction. Typically, synchrony can be exhibited across multiple communication modalities, such as through vocal patterns (Nasir *et al.*, 2018), hand and head motions (Lee *et al.*, 2014; Xiao *et al.*, 2013), and facial expressions (Guha *et al.*, 2018).

Since interpersonal synchrony in dyadic conversations provides insights toward understanding behavioral dynamics, it can potentially aid in scientific and clinical studies of interactions in the domain of ASD, which is characterized by differences in social communication and interaction (Bone *et al.*, 2016; Bone *et al.*, 2012). Prior work related to behavioral synchrony in the ASD domain is somewhat limited, focusing largely on individual modalities, such as vocal prosody or facial movements. Bone *et al.* (2014b) investigated synchrony in vocal arousal patterns in ASD child–clinician (adult) interactions, and showed its variation based on the child's ASD severity levels. To understand multimodal synchrony patterns, it is also important to consider the coordination and interplay between the communication modalities within an individual, in addition to across individuals. For example, Guha *et al.* (2018) have reported the role of localized dynamics between different facial regions and their movements, and differences therein between typically developing children and children with high functioning autism.

In this paper, we investigate three distinct measures of behavioral synchrony in speech and language patterns in an interaction based on DTW distance (Guha *et al.*, 2018), cosine distance (Nasir *et al.*, 2017; Yang and Narayanan, 2016), and word mover's distance (Nasir *et al.*, 2019). The primary contribution of this work is in quantifying synchrony across different information modalities related to voice, articulation, and language through the joint consideration of prosody, acoustic spectral features, and language patterns to capture interaction synchrony. Experiments performed on data from real world clinical interactions show that the proposed measures capture coordination in dyadic interactions. Since individuals with ASD exhibit wide differences in social communication, we believe that these coordination features can offer additional objective measures for behavior characterization and further stratification. Importantly, we experimentally investigate whether coordination features across the speech and language communication channels can capture complementary information that can be used as an additional source of information in characterizing ASD individuals and distinguishing them from those that have not received an ASD diagnosis.

We analyze differences in the synchrony measures across children with and without an ASD determination through *post hoc* classification experiments. Classification experiments carried out with the three proposed coordination features reveal their importance in differentiating ASD and non-ASD groups through improved (*F*1 score) performance with respect to baseline classifiers. Furthermore, we analyse the variation of the mean value of the proposed coordination measures throughout the interactions across two different subtasks for both children diagnosed with ASD and without ASD. We also examine age-dependency in these results through two-way analysis of variance of the classification *F*1 scores computed across three different age groups of young (2.5–7.5 y), middle-band (7.5–10 y), and older children (above 10 y), as well as male and female children.

## 2. Dataset description

The vocal and language behavioral synchrony measures are evaluated in the context of interactions between a child and a clinician. The data are drawn from two specific domains (*Emotions* and *Social difficulties and annoyance* subtasks) involving behavioral observation.

The *Autism Diagnostic Observation Schedule (ADOS-2)* (Lord *et al.*, 2000) instrument refers to semi-structured interactions between a child and a clinician trained to score the different behaviors associated with ASD. These interactive sessions are typically 40–60 min long and broken down into a variety of subtasks (e.g., construction, joint-interactive play, creating a story, demonstration, etc.) which are likely to evoke prominent response from a child under different social circumstances. Based on the child's response, the clinician provides assessment of ASD symptoms following module-specific coding and finally, all these codes are aggregated to compute an autism severity score (Gotham *et al.*, 2009).

For this study, we focus on a subset of data from the administrations of module-3 meant for verbally fluent children. Specifically, we choose to work with *Emotions* and *Social difficulties and annoyance* subtasks because of their ability to elicit spontaneous speech from the children under significant cognitive demand. The dataset consists of recordings from 165 children (86 ASD, 79 non-ASD), collected from two different clinics: the University of Michigan Autism and Communication Disorders Center (UMACC) and the Cincinnati Children's Medical Center (CCHMC). For our experiments, we have 1 recording for each of the mentioned subtasks from each participant resulting in 330 recordings. The demographic details are presented in Table 1. The average duration of each session is about 3 min (192 s). The lexical features are extracted based on manual transcriptions following SALT (Miller and Iglesias, 2012) guidelines. Since we aggregate turn-level coordination measures, sessions with fewer than 10 turns are discarded as a sufficient number of turns is required to aggregate and average out local irregularities.

**Table 1.** Demographic details of ADOS dataset.

Category	Statistics
Age (y)	Range: 3.58–13.17 (mean, std): (8.61, 2.49)
Gender	123 male, 42 female
Non-verbal IQ	Range: 47–141 (mean, std): (96.01, 18.79)
Clinical diagnosis	86 ASD, 42 ADHD
	14 mood/anxiety disorder
	12 language disorder
	10 intellectual disability, 1 no diagnosis
Age distribution clinicwise	Cincinnati 84 (2.5–7.5 y: 28, 7.5–10 y : 31, ≥ 10 y: 25)
	Michigan 81 (2.5–7.5 y: 24, 7.5–10 y: 30, ≥ 10 y: 27)
Age distribution ASD/Non-ASD	ASD 86 (2.5–7.5 y: 25, 7.5–10 y : 30, ≥ 10 y: 31)
	Non-ASD 79 (2.5–7.5 y: 27, 7.5–10 y: 31, ≥ 10 y: 21)

## 3. Quantification of interpersonal synchrony

In this section, we describe the different signal feature descriptors used and the proposed coordination measures in detail. First, we outline the feature descriptors and based on those features, we define the coordination measures. For this study, we consider three different sets of feature descriptors: vocal prosodic features, acoustic spectral features, and lexical features. We use DTWD and SCDC for vocal prosodic and acoustic spectral features to quantify interpersonal synchrony. For lexical features, we apply WMD to capture behavioral synchrony.

### 3.1 Acoustic spectral and vocal prosodic features

All the features are extracted using the OpenSMILE toolkit (Eyben *et al.*, 2010). The feature extraction is carried out using a sliding Hamming window of 25 ms duration with an interval of 10 ms. We use 15 dimensional Mel frequency cepstral coefficients (MFCC) (Yang and Narayanan, 2016) as acoustic spectral features and pitch, intensity, jitter, and shimmer as vocal prosodic features. The prosodic features are smoothed and extrapolated over the unvoiced regions.

#### 3.1.1 DTW distance measure (DTWD)

We use the classic DTW (Myers and Rabiner, 1981) method to measure the similarity between the acoustic–prosodic features extracted from two consecutive speaker turns. This method computes the (dis)similarity between two time sequences, of possibly varying lengths, after aligning them to the maximum extent in terms of a warping distance. Herein, we employ the DTW method to compute the dissimilarity between vocal feature time series obtained from the child and clinician's speech turn-pairs. We introduce the average warping distance as a measure for interpersonal synchrony.

For two *m* dimensional time series *X* and *Y* with length *T_x_* and *T_y_*, respectively, such that $X\in R^{m\times T_{x}}$ and $Y\in R^{m\times T_{y}}$, DTW finds the (dis)similarity between these sequences by optimally aligning them. A distance matrix $D\in R^{T_{x}\times T_{y}}$ is calculated where every element *d*(*i*, *j*) denotes the Euclidean distance between the *i*th vector of *X* and *j*th vector of *Y*. Based on the distance matrix values, an optimal warping path $W=w_{1},w_{2},\ldots ,w_{H}$ yielding the overall minimum cost (distance) is found. A warping path is a mapping from *X* to *Y* tracing the elements of *W* where every element is such that $w_{h}=(i,j)\;(i\in [1,T_{x}];j\in [1,T_{y}])$, i.e., *X* can be warped to the same length as *Y* by corresponding *i*th element of *X* to *j*th element of *Y*. An optimal path is the one associated with the minimum cost where the cost is computed as the sum of absolute distances for every matched pair of indices present in the path,

$$d(W)=\sum_{h=1}^{H}D(w_{h}(1),\;w_{h}(2)).$$ (1)

Since it is a dissimilarity measure, a larger warping distance is deemed to signify lesser coordination or synchrony.

#### 3.1.2 Squared cosine distance of complexity measure (SCDC)

Prior work (Nasir *et al.*, 2017) has attempted to measure coordination between speakers in a dyadic conversation from a nonlinear dynamical system approach based on the underlying model's complexity. Following a similar approach, we analyze the complexity pattern underlying the signals observed in dyadic conversations by framing them as arising from a coupled nonlinear dynamical system. However, while Nasir *et al.* (2017) relies on computing coordination in different features separately, we measure the coordination between speakers as a whole based on all the audio features considered.

We capture the difference between vocal characteristics of the speaker (turns) by comparing the complexity underlying their prosodic and spectral feature values. We use sample entropy as the complexity measure. It is an information-theoretic measure of complexity which signifies the amount of new information introduced across a temporal sequence. Based on our definition of complexity, we hypothesize that local changes of complexity in a well-coordinated conversation will be smaller than a less well-coordinated conversation. More specifically, the distance between complexity patterns corresponding to consecutive turns should be expected to be lower in a well-coordinated conversation as compared to a randomly generated conversation. As will be shown in Sec. 4, the experimental findings are found to support this hypothesis, and confirm that a larger value of the proposed measure corresponds to lower synchrony.

To calculate the proposed measure, for a time sequence of length *M* such that $X=x_{1},x_{2},\ldots x_{M}$, a length *m* subsequence is formed as $X_{m}(i)=x_{i},x_{i+1},\ldots .,x_{i+m-1}$. Let $d(X_{m}(i),X_{m}(j))$ denote the Chebyshev distance between any two such vector pairs where i≠j. Now, if $E_{m}(r+1)$ denotes the number of vector pairs such that $d(X_{m+1}(i),X_{m+1}(j))<r$ where *r* is a predefined threshold then sample entropy *S_e_* is defined as

$$S_{e}=-\text{ln}\;\frac{E_{m+1}(r)}{E_{m}(r)}.$$ (2)

From the definition, $E_{m+1}(r)$ is always less than or equal to $E_{m}(r)$, so *S_e_* is non-negative. Smaller values of *S_e_* signify greater self-similarity across the values. Here, we consider, *m *=* *2 and r=0.25× the standard deviation of the time series.

For any two consecutive pairs of turns, first, we compute the sample entropy values for every feature, yielding two vectors *X*_1_ and *X*_2_ consisting of feature-wise complexity values corresponding to the two turns. Next, we calculate the synchrony measure *σ* as

$$\sigma ={\text{cos}}^{2}\theta_{12}={\left(\frac{X_{1}^{T}X_{2}}{\left|X_{1}|\cdot |X_{2}\right|}\right)}^{2}.$$ (3)

We hypothesize the difference between sample entropy values corresponding to a turn-pair in a well-coordinated conversation will be smaller as compared to a less well-coordinated conversation. *σ* captures this information and is therefore introduced as a coordination measure.

### 3.2 Lexical features

A word embedding is a contextually derived numerical representation in a vector space for every word. In this study, we use 768 dimensional *bidirectional encoder representations from transformer (BERT)* (Devlin *et al.*, 2018) embeddings as lexical features. We choose BERT embeddings since these embeddings incorporate context which handles polysemy and nuances better.

Here, we extract BERT embeddings (BERT BASE model with 12 transformer blocks, 12 attention heads, and hidden layer dimension 768) corresponding to each word to form the feature matrix corresponding to every turn, where each row of the matrix is an embedding corresponding to one word of the turn. Once the feature matrices corresponding to the speaker turns are obtained, WMD is computed between these feature matrices corresponding to consecutive turn-pairs.

#### 3.2.1 Word mover's distance (WMD)

*Word mover's distance (WMD)* was introduced by Kusner *et al.* (2015) as a similarity measure between two text documents by comparing between *word2vec* (Mikolov *et al.*, 2013) neural word embeddings. It is calculated as the optimum distance the embedded words in one document needs to *travel* to reach the embedded words in the other document. It can be considered as a special case of the popular transportation problem of Earth mover's distance (Rubner *et al.*, 2000).

If *b_i_* and *b_j_* are the BERT embeddings corresponding to words *w_i_* and *w_j_*, respectively, the distance between these words can be defined as

$$d(w_{i},w_{j})=||b_{i}-b_{j}|{|}_{2}.$$

If *X*_1_ and *X*_2_ are the matrices corresponding to two turns of Speaker A and Speaker B, respectively, the WMD between the turns can be expressed as

$$\mathrm{WMD}(X_{1},X_{2})=\underset{T\geq 0}{\text{min}}\sum_{i=1}^{m}\sum_{j=1}^{n}T_{ij}d(w_{i},w_{j}),$$ (4)

constrained on $\sum_{i=1}^{m}T_{ij}=\frac{1}{m}$, $\sum_{j=1}^{n}T_{ij}=\frac{1}{n}$, where *m* and *n* are the number of words in turns *X*_1_ and *X*_2_, respectively, and *T_ij_* is the associated weight. Since we are working with contextual embeddings, the same word in two different positions will have distinct embeddings, so the weights are chosen to be uniform for WMD calculation.

Similar to the previously introduced measures, it is hypothesized that a conversation with greater synchrony is likely to have a smaller average WMD compared to a less synchronized conversation.

## 4. Empirical validation of the proposed synchrony measures

In this work, the DTWD and SCDC of prosodic and spectral features are introduced as interaction coordination measures. While these methods have been previously employed to capture (dis)similarity between different time series, they have not been used together in the context of quantifying interpersonal synchrony from speech audio. It should be noted that prior work (Nasir *et al.*, 2019) has established the potential of WMD in capturing linguistic coordination based on word embeddings in dyadic conversation setting. Since the use of WMD as a viable measure for interaction coordination has already been validated in previous work, in this section, we only consider validating the usefulness of measures based on DTWD and SCDC for characterising synchrony in dyadic interactions.

We use the USC CreativeIT database (Metallinou *et al.*, 2016; the details of the dataset are described in the supplementary material^1^) for these analysis experiments. It is a publicly available multimodal database consisting of dyadic conversations portraying theatrical improvisations.

We generate approximately 2500 regular (“real”) and random turn-pairs from these interactions. Any consecutive pair of turns from the same interaction is considered as a regular turn-pair while the random pairs are generated by arbitrarily choosing two turns from two different interaction sessions not involving same speakers. Our hypothesis is that for any feature set, the synchrony should be higher in the actual turn-pairs when compared to randomly shuffled pairs. For validating our hypothesis, we design a paired t-test to compare the coordination measures across the actual and random pairs with a null hypothesis being the sample mean of those two time series are equal.

The paired sample t-test result shows that the prosodic feature synchrony is significantly higher in real turn-pairs compared to the random turn-pairs, based on DTWD (*F-statistic = 4.277, p value = 0.000 01*) and SCDC (*F-statistic = 3.705, p value = 0.000 21*). A similar trend is seen for synchrony of acoustic segmental features in terms of both DTWD (*F-statistic = 2.515, p value = 0.0119*) and SCDC (*F-statistic = 3.705, p value = 0.0002*).

## 5. Experimental results on ASD interaction datasets

In this section, we report and analyze the results of the different experiments conducted to understand the differences in the proposed synchrony measures in interactions involving children with and without an ASD diagnosis.

### 5.1 Classification experiment

The experiments in this subsection explore whether the proposed measures of interaction synchrony reveal differences between interactions involving children with an ASD diagnosis and those that do not have one. This is set up as a series of classification experiments aimed at how well the children diagnosed with and without ASD can be distinguished using the proposed synchrony measures.

For the experiments, each interaction session is partitioned into child and adult speaker turns. Once the speaker turns are defined, the turns are collected into *N* segments and the coordination measures are calculated for every such segment. Hence, for each interactive session, the classifier will be input with *N* features to predict the ASD or non-ASD output label.

**Fig. 1.**
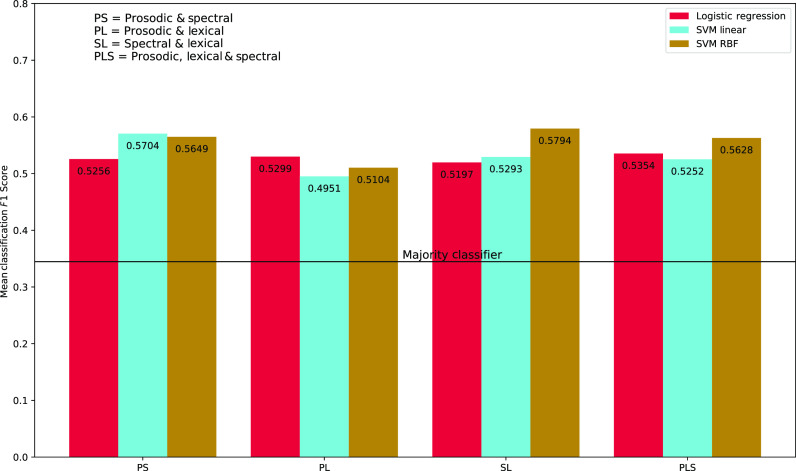
*F*1-score for ASD diagnosis with fused features.

The experiments consider three classifiers, all well established in the literature: (i) *support vector machine (SVM)* with linear kernel, (ii) SVM with *radial basis function (RBF)* kernel, and (iii) logistic regression. We also consider a classifier predicting the majority class as the baseline classifier. The classification experimental results with individual modalities are tabulated in Table 2, while those with fused features are reported in Fig. 1. We use early fusion for concatenating the features from individual modalities before feeding to the classifier.

**Table 2.** *F*1-score for ASD diagnosis.

Classifiers	Audio features				Lexical features
	Spectral features		Prosodic features		Word embeddings
	DTWD	SCDC	DTWD	SCDC	WMD
Majority Classifier	0.3446				
SVM (RBF)	0.5554	0.5345	0.5150	0.3428	0.5060
SVM (Linear)	0.5315	0.3446	0.5417	0.3448	0.4394
Logistic Regression	0.5424	0.3446	0.4865	0.3524	0.4861

### 5.2 ANOVA analysis based on age group and gender

**Fig. 2.**
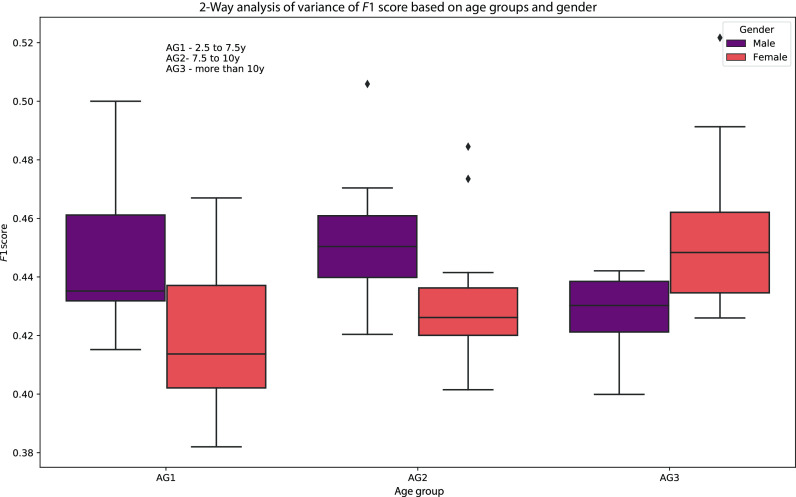
Two-way analysis of variance of *F*1 scores across age-groups and gender.

In addition to reporting the *F*1 scores for ASD/non-ASD classification experiments with the different coordination features, we also carry out a two-way analysis of variance of classification *F*1 scores across three age groups and gender. We partition the data into 3 different age groups (2.5–7.5 y, 7.5–10 y, ≥10 y) to gain insights into the synchrony features across different age groups and gender within children diagnosed with ASD and children who are not. Figure 2 presents a box plot showing the median, maximum, minimum, 75% percentile, and 25% percentile values of *F*1 scores for each age group and gender.

### 5.3 Comparison of the distribution of the proposed measures across different subtasks

**Fig. 3.**
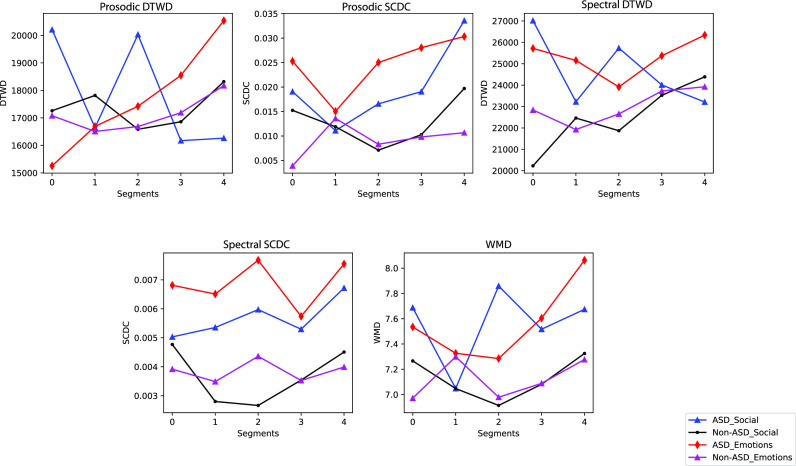
Comparison of different coordination measures across subtasks.

We also report the mean value of these coordination measures for children diagnosed with and without ASD, across the two subtasks. The comparison of the distribution of these values is shown in Fig. 3. We calculate the mean value of the coordination measures for the turns collected in five segments and plot those values across the corresponding segments.

### 5.4 Discussion

From Table 2, we observe that in the case of both of spectral and prosodic features, all classifiers yield better performance with DTWD based synchrony features compared to SCDC based features. Comparing the results from Table 2 and Fig. 1, it can be seen that fusing the synchrony features across modalities improves the classification performance over using individual modality features. Among the fused feature-based experiments reported in Fig. 1, prosodic and spectral features together show the best performance, which indicates that there is complementary information across these modalities helpful for this classification task. While all the classifiers considered provide similar performance levels, the variants of SVM with radial basis function kernel appears to be the most consistent across the experiments.

For the age group–based analysis reported in Fig. 2, the *p value = 0.000 196* suggests that the null hypothesis can be rejected implying that age difference and gender both significantly affect the classifier *F*1 scores for differentiating between children with and without ASD diagnosis based on the proposed coordination measures. Moreover, we can also find an improvement in ASD/non-ASD classification performance amongst female children in the oldest age group as compared to the other age groups. This finding stands consistent with the investigation presented in the prior work (Sturrock *et al.*, 2020) and motivates us to seek more insight to why females are more likely to go undiagnosed than males until an older age.

Figure 3 presents variation of the mean of the proposed measures across two different subtasks and ASD status. In most cases, higher mean value of these proposed measures is reported for children with an ASD diagnosis in both social and emotion subtasks. This finding indicates that children with an ASD diagnosis exhibit less synchrony in terms of these measures as compared to the children without ASD diagnosis. It is interesting to note that for all the children, emotion subtask is shown to have less synchrony for most of the duration of the session as revealed by all the measures.

## 6. Conclusion

Previous behavioral science research has established the importance of interpersonal synchrony in understanding behavior patterns in human interaction. In this work, we propose three different measures of synchrony across different aspects of speech communication (vocal acoustics, prosody, and language use). To investigate whether these synchrony features offer insights into potential differences in interaction patterns involving children diagnosed with ASD and those that do not, we set up a classification experiment utilizing the synchrony features. Results show that the proposed synchrony features can distinguish interactions involving ASD and non-ASD children indicating the role of coordination as an element of difference in social communication patterns. Moreover, the analysis shows that the synchrony features across different information modalities of spoken interactions captured by spectral features, prosodic features, and language patterns provide complementary information distinguishing the two groups: children with ASD diagnosis and without ASD diagnosis.

There are several challenging research directions to explore in future. We plan to investigate more data driven approaches to quantify synchrony instead of knowledge driven approaches. Knowing that neural networks can efficiently learn non-linear mappings between feature and coordination measures, future works can explore usage of deep neural network–based models to learn representations related to synchrony.
